# Deep Learning Reconstruction Enhances Lung Cancer CT Imaging

**DOI:** 10.7759/cureus.100762

**Published:** 2026-01-04

**Authors:** Takeshi Osaki, Akio Tamura, Shun Abe, Kunihiro Yoshioka

**Affiliations:** 1 Department of Radiology, Iwate Medical University, Yahaba-cho, JPN; 2 Division of Central Radiology, Iwate Medical University, Yahaba-cho, JPN

**Keywords:** apical lung cancer, artifact, chest wall invasion, deep learning reconstruction (dlr), noise, pancoast tumor, ultra-high-resolution ct (uhrct)

## Abstract

A 70-year-old man was referred for the surgical treatment of a right upper lobe lung adenocarcinoma. Preoperative CT revealed a tumor approximately 26 mm in size; however, the relationship between the tumor and the adjacent chest wall could not be assessed owing to noise and streak artifacts, typical of the lung apex. Ultra-high-resolution CT (UHRCT) was performed using an Aquilion Precision scanner (Canon Medical Systems; 1,792 channels per row, 0.25 mm × 160 rows, 1,024 matrix) to improve diagnostic accuracy. Images were reconstructed at 0.25-mm slice thickness using a deep learning-based reconstruction (DLR). Compared with conventional filtered back projection, the DLR images demonstrated markedly reduced noise and streak artifacts from the shoulder and clavicle, substantially improving image quality. On mediastinal window settings, tumor invasion into the superior chest wall was visualized. We thus inferred surgical resection as inappropriate; therefore, systemic chemotherapy was selected. This case demonstrates that UHRCT combined with DLR is useful for evaluating apical lung tumors that are difficult to assess using conventional CT. High-quality images provided clearer delineation of the relationship between the tumor and adjacent structures, contributing to treatment planning. DLR is a promising diagnostic approach for anatomically challenging regions such as the lung apex.

## Introduction

Superior sulcus tumors, prone to invading the thoracic inlet, may manifest diverse clinical symptoms owing to their extension to the upper ribs, thoracic vertebrae, subclavian vessels, brachial plexus, and stellate ganglion. Tumors extending beyond the parietal pleura are Pancoast tumors [1], and the involvement of complex anatomical structures makes surgical resection technically challenging [2]. As this region critically affects staging and treatment planning, preoperative imaging is vital for determining surgical indications. However, CT evaluation of the lung apices is often compromised by beam hardening and streak artifacts caused by high-density structures, such as the clavicle and scapula, and conventional filtered back projection (FBP) or hybrid iterative reconstruction (hybrid IR) provides insufficient diagnostic performance. Deep learning reconstruction (DLR) enables noise reduction and high-speed reconstruction, while preserving high spatial resolution [3]. Moreover, ultra-high-resolution CT (UHRCT) with a 0.25-mm slice thickness and a 1,024 × 1,024 matrix provides detailed imaging; however, high-performance reconstruction algorithms are required. Herein, we report a case where the combined use of UHRCT and DLR facilitated accurate evaluation of chest wall invasion in a superior sulcus tumor and substantially influenced the treatment plan.

## Case presentation

A 70-year-old man presented with digital clubbing identified during orthopedic evaluation for chronic bilateral knee pain. He was a long-term smoker (20 cigarettes per day for 50 years; Brinkman Index 1000) with an Eastern Cooperative Oncology Group performance status of 1. Screening chest CT identified a 26-mm solid mass in the right lung apex. Initial staging was cT1cN0M0 (Stage IA3), and surgical resection was planned. However, on standard FBP reconstruction, evaluation of tumor invasion into the chest wall was obscured by streak artifacts from the shoulder girdle. Therefore, UHRCT was performed (Aquilion Precision, Canon Medical Systems, Otawara, Japan; 100 kVp, AEC SD25@1 mm, rotation 0.35 s, pitch 0.994, 0.25 × 160-mm collimation, 1,024 matrix) and reconstructed using FBP (FC13), Hybrid IR (Adaptive Iterative Dose Reduction 3D: AIDR 3D Weak), and DLR (Advanced intelligent Clear-IQ Engine: AiCE, Body Standard) at 0.25-mm slice thickness. The same raw data were used for all reconstructions. Intravenous contrast enhancement was obtained using iopamidol (Iopamiron 370, 60 mL) administered at 4.0 mL/s. Radiation dose indices were CTDIvol 8.30 mGy and DLP 379.20 mGy·cm.

**Figure 1:**
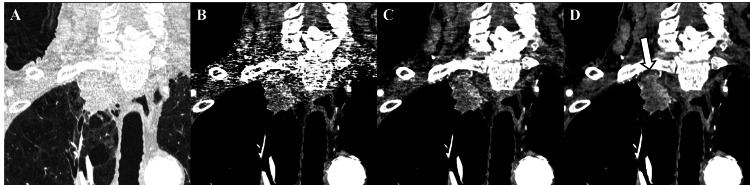
Comparison of reconstruction methods for an apical lung adenocarcinoma using ultra-high-resolution computed tomography (UHRCT). Coronal images reconstructed under the lung window setting (A) and mediastinal window settings (B–D). (A) Hybrid iterative reconstruction (Adaptive Iterative Dose Reduction 3D (AIDR 3D)), (B) filtered back projection (FBP), (C) AIDR 3D, and (D) deep learning reconstruction (Advanced Intelligent Clear-IQ Engine (AiCE)). Compared with FBP and AIDR 3D, AiCE markedly reduces streak and beam-hardening artifacts from the clavicle and shoulder, providing clearer visualization of tumor invasion into the chest wall (white arrow).

On comparison of reconstructed images (Figure 1), in the DLR images, linear artifacts were markedly reduced, enabling clear visualization of the tumor invasion into the subpleural fat and the arterial supply from the pleura to the tumor. The boundary with the subclavian artery was well delineated, showing loss of intervening fat and slight compression, both suggestive of vascular invasion. Compared with Hybrid IR, DLR further reduced image noise, clarified tumor margins, and demonstrated invasion into the subpleural adipose tissue.

**Figure 2:**
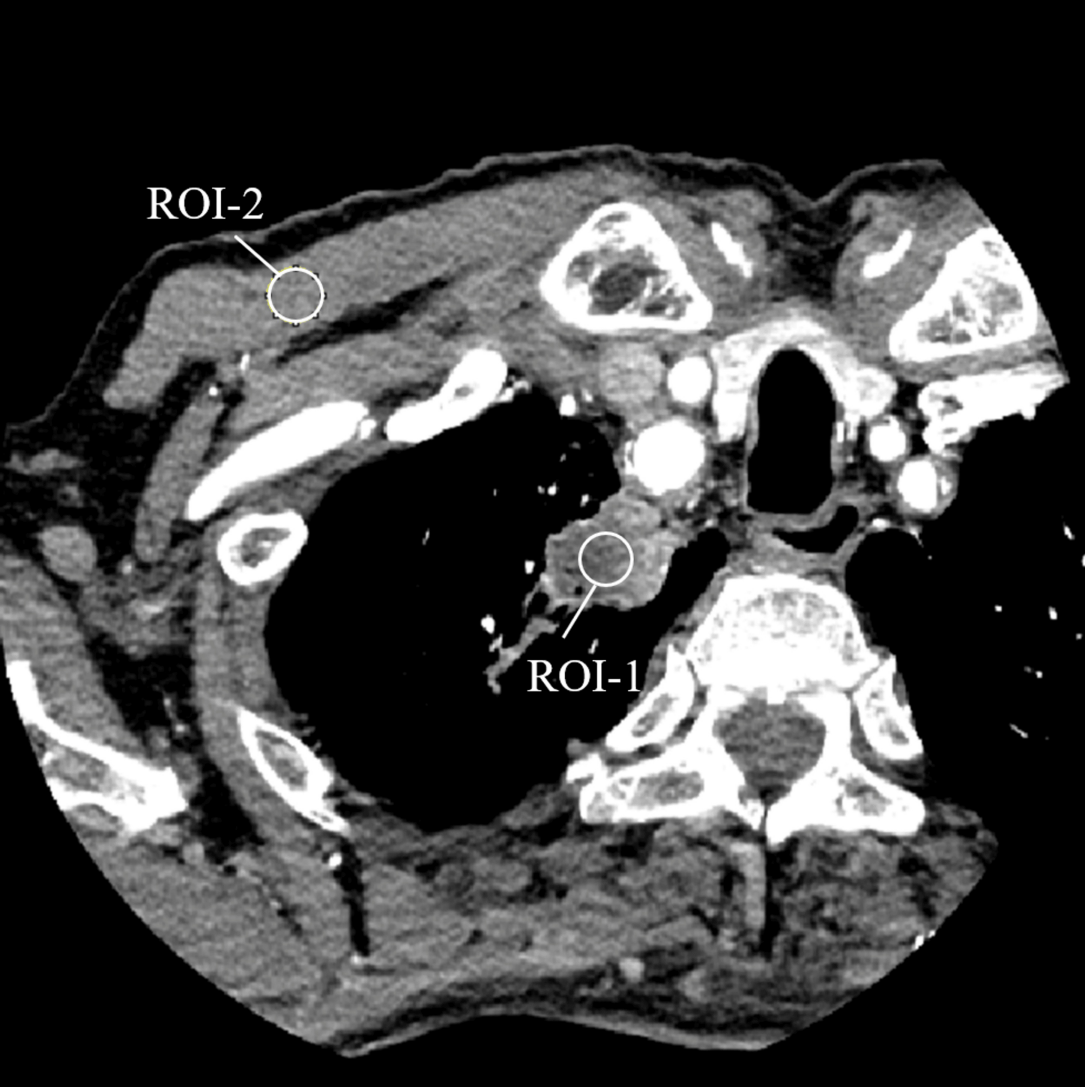
Placement of regions of interest (ROIs) for quantitative analysis. Image demonstrating 10-mm diameter circular ROIs used to measure image noise (standard deviation, SD) and contrast-to-noise ratio (CNR). ROI-1 was placed within the tumor, and ROI-2 within the pectoralis major muscle. Measurements were obtained over three contiguous axial slices (index slice + one superior/inferior slice) and averaged. CNR was calculated as (Mean_ROI-1 – Mean_ROI-2)/SD_ROI-2.

Quantitative image-quality analysis was performed using 10-mm circular regions of interest (ROIs) placed within the tumor and the pectoralis major muscle (Figure 2). DLR showed marked noise reduction compared with FBP and AIDR 3D (SD: tumor 67.9 vs. 40.2 vs. 18.6; muscle 59.3 vs. 35.9 vs. 15.3) and improved CNR (0.4 vs. 0.6 vs. 1.4), corresponding to clearer visualization of the tumor-chest wall interface (Table 1). All reconstructions were performed using the same raw projection data.

**Table 1:** Quantitative comparison of CT attenuation values, image noise, and CNR across reconstruction methods. FBP: filtered back projection; Hybrid IR: hybrid iterative reconstruction; DLR: deep learning reconstruction; ROI: region of interest; SD: standard deviation; CNR: contrast-to-noise ratio; CT value: mean attenuation value (HU); ROI-1: tumor; ROI-2: pectoralis major muscle

	CT value		SD		CNR
	ROI-1	ROI-2	ROI-1	ROI-2	
FBP	86.6	64.2	67.9	59.3	0.4
Hybrid IR	87.6	65.0	40.2	35.9	0.6
DLR	80.9	58.9	18.6	15.3	1.4

After the staging revision based on DLR findings, the case was discussed at a multidisciplinary tumor board. No MRI or other cross-sectional follow-up imaging was performed. Surgical resection was cancelled, and systemic immune checkpoint inhibitor therapy was initiated. Clinical response evaluation is ongoing.

## Discussion

In this case, chest wall invasion of an apical lung cancer, which is typically difficult to evaluate using conventional reconstruction techniques, was visualized using the combination of UHRCT and DLR. Among non-small-cell lung cancers, 5-8% invade the parietal pleura or chest wall structures, significantly influencing staging and treatment planning [4]. However, as the lung apices overlap with the clavicle and scapula, artifacts are frequently observed [5], and evaluating chest wall or brachial plexus invasion becomes challenging. CT is superior for assessing rib or vertebral erosion and vascular invasion, whereas MRI is useful for evaluating invasion of the brachial plexus, neural foramina, and spinal canal [6]. The presence or absence of chest wall invasion is critical for determining surgical eligibility, necessitating improved diagnostic performance. Uota et al. reported larger tumor size or greater contact area with the chest wall as predictive factors for chest wall invasion, indicating a higher risk of invasion [7]. Kumar et al. reported loss of the extrapleural fat plane and an obtuse tumor-pleura or tumor-chest wall interface angle as additional indicators [8]. In Pancoast tumors, MRI is particularly useful for defining the extent of local invasion and can more accurately assess pleural, subpleural fat, subclavian vessels, brachial plexus, and vertebral involvement than CT (sensitivity, 88%; specificity, 100%) [1]. Furthermore, ultrasound (US) is effective in detecting chest wall invasion, with 89% sensitivity and 95% specificity [4]. The lung apex, in close contact with the pleura in multiple directions and with limited respiratory motion, is prone to adhesions and thus at higher risk of invasion. The combination of UHRCT and DLR may enhance the visualization of chest-wall invasion and potentially influence management decisions in apical lung cancer. Nevertheless, further validation and comparison with MRI remain necessary. UHRCT provides higher spatial resolution and reduced artifacts, proving useful for imaging the lungs, coronary arteries, and peripheral arteries [9]. However, owing to its smaller detector elements and relatively limited photon flux, UHRCT tends to exhibit higher image noise than conventional CT, leading to reduced low-contrast detectability. Achieving equivalent detectability typically requires approximately 23% higher radiation dose. To overcome this issue, DLR algorithms have been developed to effectively suppress noise while maintaining image quality without increasing radiation exposure [10]. In conventional FBP, increased noise at low doses has led to the development of iterative reconstruction (IR) techniques. Among them, model-based IR (MBIR) provides excellent noise reduction and spatial resolution but requires long reconstruction times.

Recently, DLR has emerged as a deep learning-based reconstruction technique trained on paired high-dose MBIR and low-dose images to distinguish true signals from noise and artifacts. DLR yields less noise, a higher contrast-to-noise ratio, and superior subjective image quality compared with FBP and MBIR in abdominal CT [3]. Tamura et al. noted that DLR prioritizes noise suppression over artifact reduction, and its performance may depend on the characteristics of the training data. DLR achieves superior noise reduction and edge sharpness to conventional techniques; in this case, it enabled clear visualization of chest wall invasion that was indistinct with FBP. Recent evidence supports the potential of DLR to markedly improve image quality in thoracic CT. For instance, Hamabuchi et al. demonstrated that on high-definition chest CT (HDCT), DLR significantly reduced image noise and increased SNR of lung parenchyma compared with hybrid-type IR, across standard-, reduced- and ultra-low-dose protocols, and improved detection performance for various lung textures [11]. These findings align with those of our case, where DLR enabled clear delineation of the tumor-chest wall interface in an apical lung tumor, a region often compromised due to artifacts on conventional reconstructions. The presence of chest wall invasion is a key determinant of surgical eligibility. Without DLR, this case may have been deemed resectable preoperatively, only to be found inoperable at thoracotomy. The use of DLR thus allowed for the avoidance of unnecessary surgery and facilitated appropriate transition to systemic therapy, underscoring its significant clinical impact. Jensen et al. and Tamura et al. reported improved image quality and dose reduction using DLR in abdominal CT [12,13], and Uota et al. demonstrated that combining UHRCT with DLR improves the detectability of chest wall vessel invasion [7]. However, few reports have demonstrated improved diagnostic accuracy for apical lung tumors. This case is rare and, therefore, noteworthy, where DLR enhanced diagnostic accuracy and altered treatment decisions in an anatomically challenging, artifact-prone region. In cardiovascular and abdominal imaging, the effectiveness of super-resolution deep learning reconstruction (SR-DLR) has been reported [13,14]. Tamura et al. reported that SR-DLR provided superior noise texture and spatial resolution compared with conventional DLR, offering a promising balance between radiation dose reduction and diagnostic performance in low-dose CT [13]. Although data on SR-DLR on pulmonary imaging are lacking, improved visualization of lesions and chest wall invasion in artifact-prone regions, such as the lung apex, is expected in the future. This case involved a standard radiation dose; however, the excellent noise-reduction capability of DLR suggests that comparable diagnostic performance can be maintained even at lower doses. This advantage would benefit lung cancer screening and longitudinal follow-up of older adult patients.

A major limitation of this report is the absence of confirmatory histopathology or subsequent cross-sectional imaging, including MRI, to verify chest-wall invasion. The treatment decision was based solely on imaging plausibility derived from DLR. Immune checkpoint inhibitor therapy has since been initiated, and radiologic and clinical response evaluation is underway. Future studies should include larger cohorts to validate DLR’s diagnostic capability for assessing chest wall invasion, evaluate inter-vendor algorithmic variability, and standardize subjective and objective image-quality assessments.

## Conclusions

In this case, UHRCT with DLR was effective in evaluating an apical lung tumor that was difficult to assess using conventional CT. High-quality images enabled clear delineation of the relationship between the tumor and adjacent structures, directly contributing to treatment planning. DLR is a promising diagnostic tool for improving visualization in anatomically challenging regions such as the lung apex.
